# Magnetic resonance imaging of craniovertebral structures: clinical significance in cervicogenic headaches

**DOI:** 10.1007/s10194-011-0387-4

**Published:** 2011-09-27

**Authors:** Heidi Knackstedt, Jostein Kråkenes, Dalius Bansevicius, Michael Bjørn Russell

**Affiliations:** 1 Department of Neurology, Innlandet Hospital Trust, 2418 Elverum, Norway; 2 Head and Neck Research Group, Research Centre, Akershus University Hospital, Lørenskog, 1478 Oslo, Norway; 3 Department of Radiology, Section for Neuroradiology, Haukeland University Hospital, University of Bergen Medical School, 5021 Bergen, Norway; 4 Campus Akershus University Hospital, University of Oslo, 1474 Nordbyhagen, Norway

**Keywords:** Cervicogenic headache, Alar ligaments, Transverse ligaments, Craniovertebral junction, Cervical junction, MRI

## Abstract

This paper aims to investigate the relevance of morphological changes in the main stabilizing structures of the craniocervical junction in persons with cervicogenic headache (CEH). A case control study of 46 consecutive persons with CEH, 22 consecutive with headache attributed to whiplash associated headache (WLaH) and 19 consecutive persons with migraine. The criteria of the Cervicogenic Headache International Study Group (CHISG) were used for diagnosing CEH; otherwise the criteria of the International Classification of Headache Disorders (ICHD II) were applied. All participants had a clinical interview, and physical and neurological examination. Proton weighted magnetic resonance imaging (MRI) of the craniovertebral junction, and the alar and transverse ligaments were evaluated and blinded to clinical information. The MRI of the craniovertebral and the cervical junctions, the alar and transverse ligaments disclosed no significant differences between those with CEH, WLaH and or migraine. The site of CEH pain was not correlated with the site of signal intensity changes of the alar and transverse ligaments. In fact, very few had moderate or severe signal intensity changes in their ligaments. MRI shows no specific changes of cervical discs or craniovertebral ligaments in CEH.

## Introduction

Cervicogenic headache (CEH) is a symptomatic headache characterized by chronic unilateral headache possibly secondary to dysfunction of the cervical spine [1–3]. CEH is often worsen by neck movement, sustained awkward head position, external pressure of the upper cervical or occipital region on the symptomatic side [1, 2]. Anaesthetic blockades of cervical structures or related nerves can temporarily abolish pain in CEH patients, which may suggest that the pain could be attributed to a neck disorder or structural lesion [1, 2, 4]. Clinical and/or imaging evidence of neck disorder or lesion can be accepted as a valid cause of headache. However, there is an agreement that degenerative changes in the cervical spine do not necessarily correlate with pain [1, 5]. Nevertheless, the research is striven to identify causative changes in the cervical spine, which may be attributed to CEH. The craniovertebral junction is stabilized by joint capsules, tectorial membrane, transverse and alar ligaments. Those anatomic structures are innervated by C2 root [6]. Convergence of the nociceptive afferents of the trigeminal and upper three cervical spinal nerves onto the second-order neurons in the trigemino-cervical nucleus in the upper cervical spinal cord referrers the pain from the cervical spine to the head [7, 8]. The pain in CEH may originate from various anatomic structures in the cervical spine. A German study suggests that lower cervical disc prolapse may cause CEH [9]. It is conceivable that injury to the ligamentous structures can trigger CEH. High-resolution proton density-weighted magnetic resonance imaging (MRI) can visualize structural changes of ligaments and membranes in the upper cervical spine, and it is possible to grade the severity of these structural changes [10–12]. The diagnostic value of such changes is still controversial and their relevance in CEH is unknown. The aim of our study was to examine the frequency of structural changes in the alar and transverse ligaments in persons with CEH, whiplash associated headache (WLaH) and migraine.

## Materials and methods

### Study sample

The case–control study included patients referred to a general neurological outpatient clinic (Dept. of Neurology, Innlandet Hospital, Norway). A total of 118 participants were eligible for the study, but 31 refrained from participation. Of the 87 participants, 46 had CEH, 22 had WLaH, and 19 had migraine. The participants were interviewed and examined by a neurological resident (HK). CEH was classified according to the criteria of the Cervicogenic Headache International Study Group (CHISG) requiring at least three criteria to be fulfilled, not including a Greater Occipital Nerve (GON) blockade, i.e. criterias 1a, 1a1, 1a2, 1b, 1c and/or III (Table 1), [13]. Otherwise, the criteria of the International Classification of Headache Disorders (ICHD II) were applied [1]. WhipLash was defined by an acceleration/deceleration trauma that caused flexion/extension distortion of the neck followed by pain/stiffness. Three persons (two with CEH and one with migraine) refrained from MRI due to claustrophobia and two persons with CEH were excluded due to reduced image quality, ending up with 82 participants.

**Table 1:** The diagnostic criteria of cervicogenic headache by the Cervicogenic Headache International Study Group

Major criteria	I. Symptoms and signs of neck involvement Ia. Precipitation of head pain, similar to the usually occurring one Ia (1) by neck movement and/or sustained, awkward head positioning, and/or Ia (2) by external pressure over the upper cervical or occipital region on the symptomatic side Ib. Restriction of the range of motion (ROM) in the neck Ic. Ipsilateral neck, shoulder or arm pain of a rather vague, non-radicular nature, or—occasionally—arm pain of a radicular nature II. Confirmatory evidence by diagnostic anaesthetic blockades III. Unilaterality of the head pain, without side shift
Head pain characteristics	IV. Moderate–severe, non-throbbing pain, usually starting in the neck. Episodes of varying duration, or fluctuating, continuous pain
Other characteristics of some importance	V. Only marginal effect or lack of effect of indometacin. Only marginal effect or lack of effect of ergotamine and sumatriptan. Female sex. Not infrequent occurrence of head or indirect neck trauma by history, usually of more than only medium severity
Other features of lesser importance	VI. Various attack-related phenomena, only occasionally present, and/or moderately expressed when present: (a) nausea, (b) phono- and photophobia, (c) dizziness, (d) ipsilateral “blurred vision”, (e) difficulties swallowing, (f) ipsilateral oedema, mostly in the periocular area

It is obligatory that one or more of the phenomena Ia–Ic are present

### Magnetic resonance imaging protocol and evaluation

We examined the craniovertebral junction in three orthogonal planes (Siemens Symphony, Erlangen, Germany). The persons were scanned in supine position using both the neck coil and the attachable anterior element from the head coil. Images were obtained using a fast spin-echo (SE) T2 and proton-density-weighted sequences.

### MR protocol

We did a T2-weighted series covering the whole cervical spine. Repetition time (TR) and echo time (TE) were TR/TE 3,360/103, slice thickness 3 mm without gap, number of excitation (nex) 3, matrix 276 × 512 mm and field of view (FoV) 280 × 280 mm. We did proton-weighted series of the craniovertebral junction with 1.5 mm slice thickness without gap covering the alar and the transverse ligaments in three orthogonal planes. Axial series (12 images) covered from the base of the dens upward, TR/TE 2,660/15, matrix 276 × 512, nex 5, FoV 200 × 165 mm. Coronal series (13 images) covered from anterior atlantal arch backward, TR/TE 2,870/15, matrix 271 × 512, nex 5, FoV 200 × 200 mm. Sagittal series (20 images) covering the entire length of both alar ligaments, TR/TE 2,150/15, matrix 211 × 512, nex 3, FoV 200 × 150 mm.

The classification of the alar and transverse ligament lesions is based on the ratio between any high-signal part and the total cross-sectional area of the ligament. The alar and the transverse ligaments were graded according to the following criteria: grade 0—ligament with low signal throughout the entire cross-section; grade 1—ligaments with high signal in <1/3 or less of cross-section; grade 2—high signal in 1/3–2/3 of cross-section and grade 3—high signal in >2/3 or more of cross-section. Both sides of the alar and transverse ligaments were visualized in all participants [10–12, 14].

### MRI evaluation

All MR images were evaluated by an experienced consultant in Neuroradiology (JK), who was blinded to clinical information.

### Statistical analysis

The statistical analysis was performed using SPSS Base System for Windows 15.0 for all four MRI gradings and dichotomized groups (Grade 0–1 and Grade 2–3). We used the χ^2^-test with 5% level of significance.

### Ethical issues

The Regional Committees for Medical Research Ethics and the Norwegian Social Science Data Services approved the project. The participants that received GON blockade were informed about the procedure and side effects. All participation was based on informed consent.

## Results

Table 2 shows demographic data of the participants. Signal intensity changes in the alar and transverse ligaments were found in 43% (*n* = 18) of persons with CEH, in 41% (*n* = 9) in persons with WLaH and in 50% (*N* = 9) of the persons with migraine. The results were dichotomized in two groups between none to mild (grade 0–1) and moderate to severe (grade 2–3) signal intensity changes. Table 3 shows that moderate to severe signal intensity changes in any of the transverse or alar ligaments (graded 2–3) were equally distributed on the right and left side and there were no statistical significant differences between the CEH, WLaH or migraine groups. Only 16% had moderate or severe signal changes. Mild signal intensity changes (grade 1) were found in 21, 32, and 44% of the subjects with CEH, WLaH and migraine, respectively. We disclosed no statistical significant changes regarding side of the change or between the CEH, WLaH and migraine groups dichotomizing the groups into none and mild to severe signal intensity changes (graded 0 and 1–3).

**Table 2:** Demographic data

	Cervicogenic headache *N* = 46	Whiplash associated headache *N* = 22	Migraine *N* = 19
Women (*n*)	36	13	17
Men (*n*)	10	9	2
Age mean (SD)	43.2 (9.2)	41.5 (7.1)	42.3 (11.2)
Age range (year)	27–61	27–57	21–58
Age at onset mean years (SD)	31.3 (11.9)	33.4 (9.8)	19.9 (8.1)
Headache duration mean (SD)	12.4 (10.4)	8.6 (7.1)	22.1 (11.6)

**Table 3:** Signal intensity changes in any of the transverse or alar ligaments (details for grading is described in “Materials and methods” section)

	CEH *N* = 42% (*n*)	WLaH *N* = 22% (*n*)	Migraine *N* = 18% (*n*)	*p* value
*Right alar ligament*				
Grade 0–1	86 (36)	86 (19)	89 (16)	n.s.
Grade 2–3	14 (6)	14 (3)	11 (2)	
*Left alar ligament*				
Grade 0–1	86 (36)	95 (21)	89 (16)	n.s.
Grade 2–3	14 (6)	5 (1)	11 (2)	
*Both sides alar ligament*				
Grade 0–1	83 (35)	86 (19)	89 (16)	n.s.
Grade 2–3	17 (7)	14 (3)	11(2)	
*Right transverse ligament*				
Grade 0–1	90 (38)	95 (21)	100 (18)	n.s.
Grade 2–3	10 (4)	5 (1)	0 (0)	
*Left transverse ligament*				
Grade 0–1	88 (37)	95 (21)	89 (16)	n.s.
Grade 2–3	12 (5)	5 (1)	11 (2)	
*Both sides transverse ligament*				
Grade 0–1	88 (37)	91 (20)	89 (16)	n.s.
Grade 2–3	12 (5)	9 (2)	11 (2)	

*n.s.* denotes non-significant

Table 4 shows disc degeneration. Moderate or severe degeneration of the craniovertebral and cervical discs was rare and only found in the C4/5, C5/6 and C6/7. Changes were seen in all three diagnostic groups, although there were no significant differences among the groups.

**Table 4:** Signal intensity changes in the craniovertebral and cervical junction (details for grading is described in “Materials and methods” section)

	CEH *N* = 42% (*n*)	WLaH *N* = 22% (*n*)	Migraine *N* = 18% (*n*)	p value
*C2/3*				
Grade 0–1	100 (42)	100 (22)	100 (18)	n.s.
Grade 2–3	0 (0)	0 (0)	0 (0)	
*C3/4*				
Grade 0–1	100 (42)	100 (22)	100 (18)	n.s.
Grade 2–3	0 (0)	0 (0)	0 (0)	
*C4/5*				
Grade 0–1	88 (37)	91 (20)	89 (16)	n.s.
Grade 2–3	12 (5)	9 (2)	11 (2)	
*C5/6*				
Grade 0–1	69 (29)	91 (20)	83 (15)	n.s.
Grade 2–3	31 (13)	9 (2)	17 (3)	
*C6/7*				
Grade 0–1	95 (40)	100 (22)	100 (18)	n.s.
Grade 2–3	5 (2)	0 (0)	0 (0)	
*C7/TH1*				
Grade 0–1	100 (42)	100 (22)	100 (18)	n.s.
Grade 2–3	0 (0)	0 (0)	0 (0)	
*Change any junctions*				
Grade 0–1	70 (29)	86 (19)	84 (15)	n.s.
Grade 2–3	30 (13)	14 (3)	16 (3)	

*n.s.* denotes non-significant

Signal intensity changes in the transverse and alar ligaments in relation to the location of the CEH are shown in Table 5. The statistical analyses showed no significant correlation between the site of signal intensity change and site of CEH. Dichotomizing the results in none and mild to severe signal intensity changes did not change the outcome of the analyses.

**Table 5:** Signal intensity changes in the transverse and alar ligaments in relation to location of the cervicogenic headache (CEH)

	Grade of structural changes on MRI		*p* values
	0–1	2–3	
Right-sided CEH *n* = 19	*N* (%)	*N* (%)	
Right alar ligament	17 (89)	2 (11)	n.s.
Left alar ligament	17 (89)	2 (11)	
Right transverse ligament	15 (80)	4 (20)	n.s.
Left transverse ligament	14 (74)	5 (26)	
Left-sided CEH *n* = 23			
Right alar ligament	19 (83)	4 (17)	n.s.
Left alar ligament	19 (83)	4 (17)	
Right transverse ligament	23 (100)	0	n.s.
Left transverse ligament	23 (100)	0	

*n.s.* denotes non-significant

## Discussion

We found no significant difference in MRI signal intensity changes in the alar and transverse ligaments or any difference in disc degenerative between subjects with CEH, WLaH and migraine. However, the pain in CEH may originate from various other structures in the cervical spine and cervical ligaments not identified with this MRI protocol which focused on certain structures [15]. But still all pathological changes in the cervical spine with sensory connection to the spinal tract of the trigeminal nerve might potentially be the pain generating structures which has to be focused on [7]. The alar ligament system is involved during cervical extension, lateral flexion, and ipsilateral rotation; nevertheless we found no correlation between side location of pathological signal intensity (higher signal intensity) in the ligaments and the side location of the CEH [16, 17]. The transverse ligaments are strained at various movements of the head, still high-signal intensity (graded 2–3) in those ligaments was rare in all three diagnostic groups. A cross-sectional study applying conventional cervical MRI found no significant difference between patients with CEH and control subjects [18]. More specifically designed MRI protocols and evaluation grading scales were introduced focusing on the structural assessment of craniovertebral ligaments and craniovertebral junctions in persons with whiplash associated disorders [12, 14, 19, 20]. High grade changes were far more frequently observed in cases with a previous whiplash trauma than in a control group using a high-resolution proton density-weighted MRI in three orthogonal planes [10, 11].

There are at least four case control studies that used similar MRI methodology as our present study—two of those studies suggests injury of craniocervical structures, while two recent studies failed to reproduce those findings. A new improved MRI protocol showing the ligaments and membranes in the craniovertebral junction was developed 10 years ago [14]. Further, they studied and classified structural changes in the alar ligaments in the late stage of whiplash injuries by the use of a new MRI protocol [10]. Almost half of whiplash associated disorder (WAD) subjects had structural changes in the alar ligaments, while no grade 2 or 3 lesion was found in the control group. Authors suggest that whiplash trauma might cause permanent damage to the alar ligaments, shown by high-resolution proton density-weighted MRI but the reliability of this classification had to be improved. A similar study has been performed by the same group focusing on MRI changes of the tectorial and posterior atlanto-occipital membranes [11]. A study on the radiologic spectrum of craniocervical distraction injuries used fat suppressed T2 weighted images a method that might be more sensitive to demonstrate increased signal intensity in the atlantoaxial and atlanto-occipital joints, craniocervical ligaments, prevertebral soft tissue and spinal cord than conventional MRI, however, we used a specific MRI protocol developed with special emphasis on imaging the ligaments [14, 21]. Those studies triggered lively discussion between neurologists and radiologists and there was a need of similar studies from other groups that could confirm diagnostic value of those MRI techniques. Myran et al. [20] compared subjects with WAD, chronic non-traumatic neck pain and subjects without neck pain or previous neck trauma. Alar ligament changes grade 0 to 3 were seen in all three groups. Areas of high-signal intensity (grade 2–3) were found in at least one alar ligament in 49% of the patients in the whiplash associated disorder grade I–II group, in 33% of the chronic neck pain group and in 40% of the control group. The diagnostic value and the clinical relevance of magnetic resonance detectable areas of high intensity in the alar ligaments are questionable. Another study examined ligaments and membranes in the craniocervical junction with MRI in patients with WAD and compared them with healthy control subjects [22]. High-signal intensity of the alar and transverse ligaments was quite common and was reported at an average of about 50% both among patients and control subjects. The incidence of abnormalities of the tectorial and posterior atlanto-occipital membranes was low in both groups. No statistically significant difference between control subjects and patients with WAD was revealed for any of the structures assessed.

Our study failed to show differences or specific changes of cervical discs or craniovertebral ligaments in any studied group. However, our primary focus was somehow different compared with other similar studies. CEH is a defined headache syndrome, while WLaH or WAD could be defined by different symptoms and only one thing in common—neck trauma in the past. Unilaterality of symptoms in CEH allowed us to look for MRI changes at corresponding side.

Structural alterations of the alar ligaments and upper articular joints are frequent in asymptomatic patients [19]. Focussing on only one particular structural change in the cervical spine might not be a suitable diagnostic method to detect possible pathological finding in patients with CEH. Future investigations might have to focus more on the heterogenic origin of CEH and alternative operational tests in addition to the MRI.

## Conclusion

Morphological MRI changes in craniovertebral ligaments showed similar frequency in patients with CEH compared to those with WLaH and/or migraine. According to our data, such changes have no established value for the diagnosis or work up of CEH.
